# Research on Bearing Fault Diagnosis Method Based on Filter Features of MOMLMEDA and LSTM

**DOI:** 10.3390/e21101025

**Published:** 2019-10-22

**Authors:** Yong Li, Gang Cheng, Xihui Chen, Yusong Pang

**Affiliations:** 1 School of Mechatronic Engineering, China University of Mining and Technology, Xuzhou 221116, China; 2 College of Mechanical and Electrical Engineering, Hohai University, Changzhou 213022, China; 3 Faculty Mechanical, Maritime and Materials Engineering, Delft University of Technology, 2628 Delft, The Netherlands

**Keywords:** bearing, fault diagnosis, MOMLMEDA, filter feature, LSTM

## Abstract

As the supporting unit of rotating machinery, bearing can ensure efficient operation of the equipment. Therefore, it is very important to monitor the status of bearings accurately. A bearing fault diagnosis mothed based on Multipoint Optimal Minimum Local Mean Entropy Deconvolution Adjusted (MOMLMEDA) and Long Short-Term Memory (LSTM) is proposed. MOMLMEDA is an improved algorithm based on Multipoint Optimal Minimum Entropy Deconvolution Adjusted (MOMEDA). By setting the local kurtosis mean as a new selection criterion, it can effectively avoid the interference of false kurtosis caused by noise and improve the accuracy of optimal kurtosis position. The optimal filter designed by optimal kurtosis position has periodic and amplitude characteristics, which are used as the fault feature in this paper. However, this feature has temporal characteristics and cannot be used as input of general neural network directly. LSTM is selected as the classification network in this paper. It can effectively avoid the influence of the temporal problem existing in feature vectors. Accurate diagnosis of bearing faults is realized by training classification neural network with samples. The overall recognition rate is up to 93.50%.

## 1. Introduction

Bearing is an important part of mechanical equipment. It can reduce the friction coefficient and ensure its rotary accuracy in the process of motion. Bearing failures occur from time to time under long-term, high-speed, and heavy-load conditions, seriously affecting the overall operation efficiency of the equipment. Effective health monitoring of bearing and reasonable arrangement of maintenance and replacement are of great significance to improve the overall production efficiency [1]. Fault diagnosis technology based on vibration signals is widely used in bearing health monitoring. Vibration signals can effectively respond to impulses generated by bearing faults and other characteristics [2]. However, due to noise interference, false impulses often occur [3]. Therefore, the research on eliminating false impulse interference is very important to ensure the accurate diagnosis of bearing faults.

Kurtosis is a numerical statistic that can reflect the distribution characteristics of random variables [4]. It is especially sensitive to impulse signals in fault diagnosis. Using this feature, R.A. Wiggins proposed Minimum Entropy Deconvolution (MED) in 1978 and applied it to seismic records [5]. MED can effectively extract impulse components from signals [6]. In order to make up for the limitation of MED, McDonald and others first proposed Maximum Correlated Kurtosis Deconvolution (MCKD) [7]. MCKD introduces the concept of correlation kurtosis and takes maximizing correlation kurtosis as the objective function, which can effectively extract periodic impulses [8]. Lyu proposed an improved method based on quantum genetic algorithm (QGA) named QGA-MCKD [9]. Two key parameters, filter length and deconvolution period of MCKD, correspond to each single fault and are adaptively selected by QGA. It is effective in bearing compound fault diagnosis. However, MCKD can only obtain a single failure cycle. MCKD becomes powerless when the signal contains multiple failure cycles. McDonald and others further proposed Multipoint Optimal Minimum Entropy Deconvolution Adjusted (MOMEDA) [10]. MOMEDA proposes a deconvolution problem aiming at infinite impulse sequence, which can directly calculate the optimal filter solution and effectively avoid the iteration problem [11]. Zhou proposed a parameter adaptive MOMEDA method based on grasshopper optimization algorithm to extract fault features [12]. The introduction of GOA not only solves the problem of parameter selection in MOMEDA, but also achieves better performance compared with other optimization methods. The feasibility and superiority of the approach are fully proved.

However, due to the influence of noise, false peaks often occur in the kurtosis spectrum, which interferes with the selection of the optimal kurtosis location [13]. This makes some filters designed based on the selected position have essential errors. In order to eliminate the interference of noise, an improved algorithm called MOMLMEDA is proposed in this paper. In this method, the maximum local mean is selected as a new criterion of the optimal location. The influence of false peaks can be effectively solved by utilizing the large width characteristic of real maximum kurtosis.

A good fault feature can effectively represent different fault signals [14]. It not only needs to reflect the difference between the different kinds of signals, but also needs to ensure the similarity between the same kind of signals [15]. The filter vector based on the optimal kurtosis position can effectively reflect the periodicity and amplitude characteristics of the original fault impulse signals [16]. It can be used as a fault feature to detect bearing health effectively. There are many traditional classification methods such as support vector machines, quadratic classifiers, back-propagation neural networks [17]. Jovan Gligorijevic proposed a bearing fault diagnosis method based on wavelet transform and quadratic classifier. The new technique can increase reliability and efficiency in the industry by preventing unexpected faulty operation of bearings [18]. However, there are some temporal differences in the feature distribution per unit time, which affect the stability of the feature vector to a certain extent [19]. It is difficult for a traditional classifier to achieve better classification results under the influence of temporal characteristics. In order to eliminate the influence of temporal characteristics on feature vectors, Long Short-Term Memory (LSTM) neural network is selected as the classification network. LSTM is a kind of Recurrent Neural Network (RNN), which is used to process sequential data [20]. It can effectively establish the weight connection between neurons in a certain layer and the internal relationship between features [21]. In addition, the input gate, the forgetting gate, and the output gate can be set in each cell to judge whether the input information is useful or not according to the rules. Such a rule can effectively avoid the problem that the long-term memory cannot be established due to the disappearance of gradient [22]. This network has been widely used in language translation, image analysis, document abstraction, speech recognition, and so on [23] Gao proposed a novel end-to-end framework named aLSTMs, an attention-based LSTM model with semantic consistency, to transfer videos to natural sentences. It can guarantee the semantic consistence of the sentence description and the video visual content effectively. [24]. In this paper, it is applied to fault classification based on filter features. It can effectively eliminate the interference of temporal characteristics on fault characteristics and improve the stability of classification network. Finally, the neural network trained by samples can accurately diagnose the bearing health status.

## 2. Model Building

**Figure 1:**
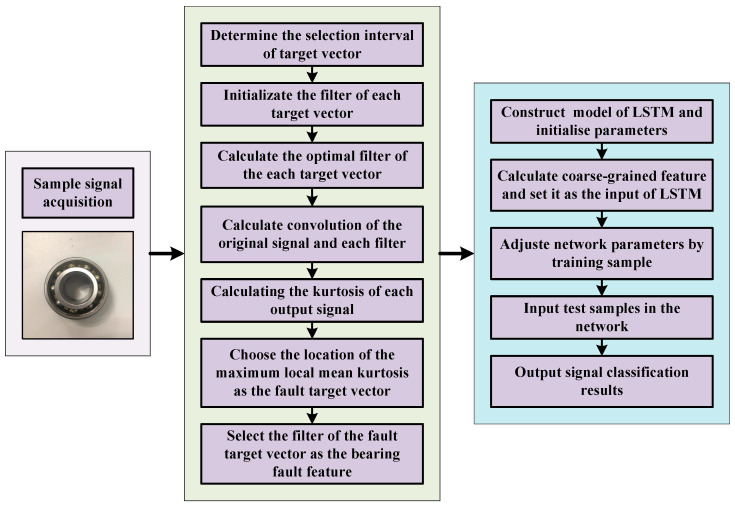
The flowchart of the proposed method.

In this paper, a bearing fault diagnosis mothed based on MOMLMEDA and LSTM is proposed. This method makes full use of the advantages of two methods in dealing with noise interference and temporal interference respectively. The flowchart of the proposed method is shown in Figure 1.

### 2.1. Coarse-Grained Feature Extraction of Filter Based on MOMLMEDA

The essence of MOMEDA algorithm is to find a filter to extract the periodic impulse component in the input signal as far as possible. The filter can effectively extract impulse components from the original signal. It has good periodic and amplitude characteristics. However, the original MOMEDA is vulnerable to false kurtosis peaks caused by noise when choosing optimal kurtosis location, and its stability is poor. MOMLMEDA takes maximum local mean as selection criterion of the optimal kurtosis position, which effectively avoids the influence of false kurtosis peak and improves the accuracy of the designed filter.

Assuming the collected vibration signal x(n) and the filter f(l), the output signal y(n) can be expressed as:

(1) y(n)=f(l)∗x(n)

The known position multiple impulses are taken as deconvolution targets. This maximization problem is called MOMEDA.

(2) $$\mathrm{Multi}\;D-\mathrm{Norm}=MDN(y,t)=\frac{1}{\left\|t\right\|}\frac{t^{T}y}{\left\|y\right\|}$$

(3) $$\underset{f}{\max}MDN(y,t)=\underset{f}{\max}\frac{t^{T}y}{\left\|y\right\|}$$

where t is a constant vector that defines the locations and weightings of a series of goal impulses.

The extremum of the above formula is obtained by derivation:

(4) $$\frac{d}{d_{f}}\left(\frac{ty}{\left\|y\right\|}\right)=\frac{d}{d_{f}}\frac{t_{1}y_{1}}{\left\|y\right\|}+\frac{d}{d_{f}}\frac{t_{2}y_{2}}{\left\|y\right\|}+\cdots +\frac{d}{d_{f}}\frac{t_{N-L}y_{N-L}}{\left\|y\right\|}$$

The derivation of each single item on the right side of the Equation (4) is as follows:

(5) $$\frac{d}{d_{f}}\left(\frac{t_{k}y_{k}}{\left\|y\right\|}\right)={\left\|y\right\|}^{-1}t_{k}M_{k}-{\left\|y\right\|}^{-3}t_{k}y_{k}X_{0}y$$

(6) $$M_{k}=\left|\begin{array}{c} x_{k+L-1}\\ x_{k+L-2}\\ \vdots \\ x_{k} \end{array}\right|$$

Then, the formula is written as follows:

(7) $$\frac{d}{d_{f}}\left(\frac{t_{k}y_{k}}{\left\|y\right\|}\right)={\left\|y\right\|}^{-1}X_{0}t-{\left\|y\right\|}^{-3}tyX_{0}y$$

(8) $$X_{0}t=t_{1}M_{1}+t_{2}M_{2}+\cdots +t_{N-L}M_{N-L}$$

Solving for extremum by equating to 0:

(9) $${\left\|y\right\|}^{-1}X_{0}t-{\left\|y\right\|}^{-3}tyX_{0}y=0$$

(10) $$\frac{ty}{{\left\|y\right\|}^{2}}X_{0}y=X_{0}t$$

Since $y=X_{0}^{T}f$ and assuming ${\left(X_{0}X_{0}^{T}\right)}^{-1}$ exists:

(11) $$\frac{ty}{{\left\|y\right\|}^{2}}f={\left(X_{0}{X_{0}}^{T}\right)}^{-1}X_{0}t$$

Since multiples of f are also solutions to Equation (11), multiples of $f={\left(X_{0}{X_{0}}^{T}\right)}^{-1}X_{0}t$ are solutions to the MOMEDA problem.

(12) $$f={\left(X_{0}{X_{0}}^{T}\right)}^{-1}X_{0}t$$

(13) $$X_{0}=\left|\begin{array}{ccccc} x_{L} & x_{L+1} & x_{L+2} & \cdots & x_{N}\\ x_{L-1} & x_{L} & x_{L+1} & \cdots & x_{N-1}\\ x_{L-2} & x_{L-1} & x_{L} & \cdots & x_{N-2}\\ \vdots & \vdots & \vdots & \ddots & \vdots \\ x_{1} & x_{2} & x_{3} & \cdots & x_{N-L+1} \end{array}\right|$$

MOMEDA can calculate a spectrum of *M* target vector candidates. The calculation formulas of filter vector matrix *F* and kurtosis value *MKurt* are as follows:

(14) $$F=\left[\begin{array}{cccc} f_{1} & f_{2} & \cdots & f_{M} \end{array}\right]=\left(X_{0}{X_{0}}^{T}\right)X_{0}\left[\begin{array}{cccc} t_{1} & t_{2} & \cdots & t_{M} \end{array}\right]$$

(15) $$Y=\left[\begin{array}{cccc} y_{1} & y_{2} & \cdots & y_{M} \end{array}\right]={X_{0}}^{T}F$$

(16) $$MKurt(y,t)=\frac{{\left(\sum_{n=1}^{N-L}{t_{n}}^{2}\right)}^{2}}{\sum_{n=1}^{N-L}{t_{n}}^{8}}\frac{{\sum_{n=1}^{N-L}\left(t_{n}y_{n}\right)}^{4}}{{\left(\sum_{n=1}^{N-L}{t_{n}}^{2}\right)}^{2}}$$

Sort the values in the obtained *MKurtsx.*

(17) [MKurtsx Index]=sort[MKurt]

*MKurtsx* is the sorted numeric vector, Index is the original sequence corresponding to the numeric vector, and sort is the sorting function.

Then, the first *K* positions with larger values are selected and the local mean kurtosis $MKlm_{k}$ of the positions is calculated.

(18) $$MKlm_{k}=\mathrm{mean}[MKurt_{index_{k}-n}\;MKurt_{index_{k}-n+1}\ldots \;MKurt_{index_{k}+n}]$$

mean is the mean calculation function.

Finally, the maximum kurtosis mean is selected and defined as the optimal kurtosis value.

(19) $$MK_{best}=\max(MKlm_{k}),k=1,2\ldots K$$

max represents the maximum selection function.

The location of the kurtosis value is *t_best_*, the designed filter *f_best_* based on this position is defined as the optimal one.

However, the filter length is usually large. If it is directly input into the neural network as a feature, it will lead to a larger network scale and increase the amount of computation. In order to solve this problem, the filter is coarsened. Each filter with *L* length is divided into *k* groups. The global mean of each segment is calculated separately and used as a new feature vector *S* = (*s*_1_ *s*_2_ …… *s_k_*).

(20) $$s_{k}=\frac{f_{k,1}+f_{k,2}+\cdot \cdot \cdot +f_{k,l}}{l}$$

### 2.2. Classification Network Construction Based on LSTM

**Figure 2:**
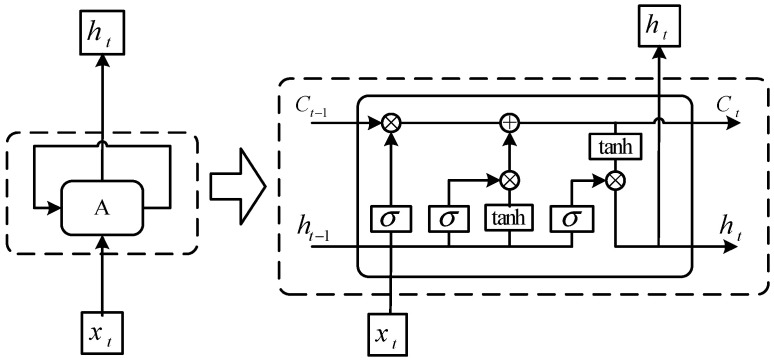
The framework of LSTM.

The classification network based on LSTM consists of five layers: input layer, LSTM layer, full connection layer, soft Max layer, and classification output layer. The LSTM layer is the core of the whole classification network. Its framework is shown in the Figure 2.

Compared with RNN, LSTM replaces internal storage with cell. Cell state runs through the entire LSTM architecture with a small amount of linear interaction, which enables the network to learn long-term dependent information. A standard LSTM layer is divided into four steps to process data.

Step 1: Determines which old information needs to be removed from the cell state by the forget gate. The forget gate is composed of sigmoid function.

(21) $$f_{t}=\sigma (W_{f}(h_{t-1},x_{t})+b_{f})$$

Step 2: Determine which new information needs to be added into cell state by input gate. The input gate is composed of a sigmoid function and a tanh function. The sigmoid function determines which values need to updated. The tanh function is used to create a new candidate value vector.

(22) $$i_{t}=\sigma (W_{i}(h_{t-1},x_{t})+b_{i})$$

(23) $${\tilde{C}}_{t}=\tanh(W_{C}(h_{t-1},x_{t})+b_{C})$$

Step 3: Update cell status based on step 1 and step 2.

(24) $$C_{t}=f_{t}*C_{t-1}+i_{t}*{\tilde{C}}_{t}$$

Step 4: Determines which information needs to be export by output gate. The output gate is composed of a sigmoid function and a tanh function. The sigmoid function determines which part of the cell state will be exported. The tanh function is used to process the cell state and convert it into a value which is between -1 and 1. The final output can be obtained by multiplying the output of the two functions.

(25) $$o_{t}=\sigma (W_{o}(h_{t-1},x_{t})+b_{0})$$

(26) $$h_{t}=o_{t}*\tanh(C_{t})$$

## 3. Experimental Data Acquisition

### 3.1. Introduction of Test Bench

**Figure 3:**
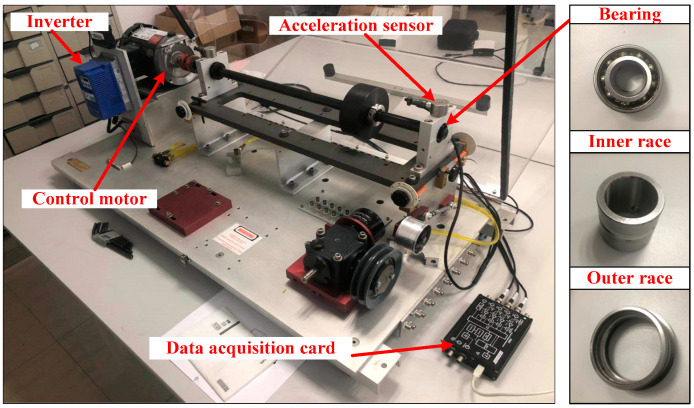
SQI-MFS bearing fault test bench.

The experimental data used in this paper is from SQI-MFS bearing fault test bench. The test bench belongs to ourselves. Data is also collected by ourselves according to the needs of experiments. The SQI-MFS test bench is composed of motor, frequency converter, bearing, accelerometer, and base bracket. It can effectively simulate the bearing faults. The type of rolling bearing used in the experiment is SER205. The different faults of bearing are complete by laser processing. The fault sizes of inner race and outer race are 0.1 mm, 0.2 mm and 0.3 mm. With normal bearings and ball fault bearing, there are eight kinds of bearing states. The test bench and the bearing are shown in Figure 3.

### 3.2. Data Acquisition Scheme

Data sample acquisition is under fixed load and rotating speed. The motor speed is 1200 r/min and the sampling frequency is 16 kHz. Eight groups of different bearing vibration signals were sampled in this experiment. Each group contains 100 samples and each sample contains 8000 sample points (0.5 s). Table 1 shows the description of classification.

## 4. Experiment Data Analysis

Figure 3 shows the time domain comparison of normal bearing signals and fault bearing signals with different fault types and degrees. The sampling time of each signal is 0.5 s and the number of sampling points is 8000.

**Figure 4:**
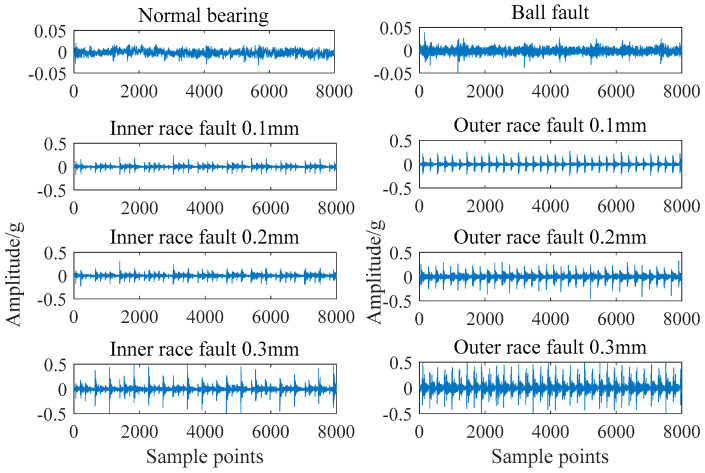
Time domain comparison of different bearing signals.

It can be seen from Figure 4 that the failure of bearings is mainly presented in the form of periodic impact. There are some differences in the periodicity and amplitude. Using this characteristic to extract a feature vector can effectively realize the diagnosis of bearing condition.

**Figure 5:**
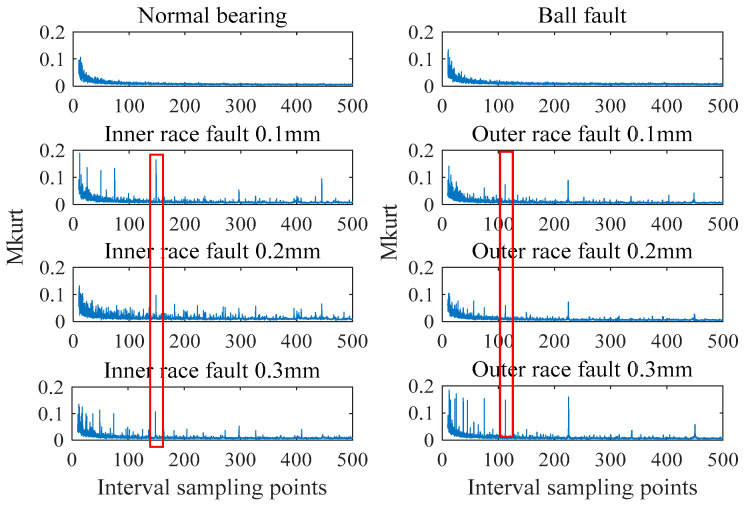
Multi kurtosis spectrum of different bearing signals.

MOMEDA is a fault feature extraction method based on the periodic characteristics of rotating machinery. It overcomes the problem of single periodic impulse in MED and the problem of non-optimal filter in MCKD. By automatically selecting the maximum kurtosis point in a fixed range and designing the optimal filter, the periodic impulse signal can be extracted. The eight signals shown in Figure 1 are processed by MOMEDA. In order to observe the kurtosis changes in a wide range, the initial range of kurtosis calculation interval is set to 10–500. Because a too-large filter length will increase operation time, the initial value of the largest length is 500. The multi kurtosis spectrum obtained after processing is shown in Figure 5.

As can be seen from Figure 5, compared with the multi-point kurtosis spectrum of the normal signal, ball failures do not show much difference, but the kurtosis spectra of inner and outer race have obvious peaks. The kurtosis peak of the inner race fault signal appears near the sampling point 148, and the kurtosis peak of the outer race fault signal appears near the sampling point 112. Kurtosis spectrum can well reflect the periodic characteristics of fault shocks. After this treatment, the inner race fault and outer race fault can be distinguished well.

Since the ideal peak positions of this set of data are 112 and 148, the calculation interval is set to 100–160 and the minimum filter length is set to 200 at least in order to make the designed filter contain the whole period. Taking 100 as step size and the accuracy of the optimal kurtosis position as criterion, the effect of filters with different lengths in the range of 200–2000 interval on the selection of optimal kurtosis peaks is tested. Since the normal signals and ball fault signals have no obvious periodic impulse component, different fault degree signals of inner race and outer race are taken to test the influence of different filter lengths on the optimal kurtosis location selection. There are six kinds of fault signals and each has 50 samples. The test result is shown in Figure 5.

**Figure 6:**
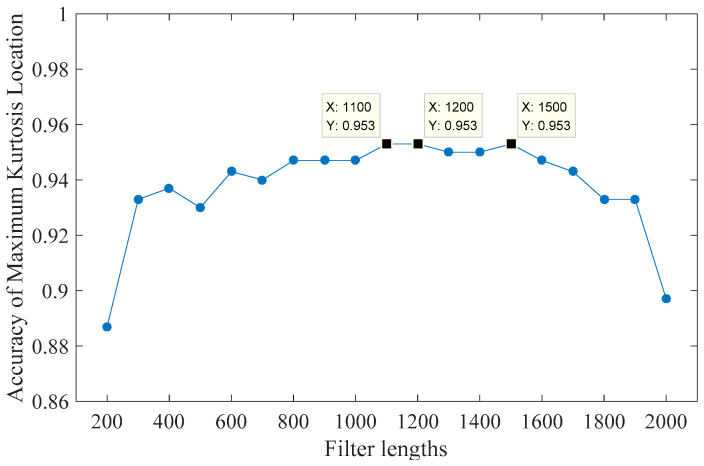
Test result under different lengths of filter via MOMEDA.

**Figure 7:**
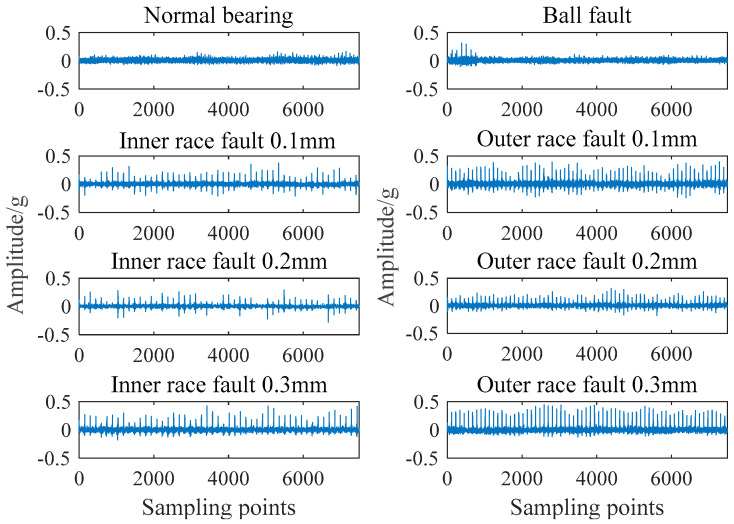
Output signals obtained by MOMEDA.

As can be seen from Figure 6, when the filter length increases from 200 to 300, the accuracy of the optimal kurtosis location selection increases significantly, but when the filter length is longer than 300, the improvement decreases sharply. When the length is 1100, the maximum accuracy is 95.3%. The output signals obtained by the convolution of the designed filter based on the maximum kurtosis position are shown in Figure 7.

It can be seen that the output signal processed by the filter can effectively eliminate the noise component contained in the signal and can effectively extract the impulse component in the original signal.

Although this peak fault feature is obvious, it is not stable enough. It was found in experiments that the selection of the optimal kurtosis location in the search interval is susceptible to the interference of false peaks caused by noise. The filter based on this kurtosis becomes meaningless. Therefore, eliminating the influence of false peak value on filter design is of great significance to the final bearing fault diagnosis. It can be seen from Figure 6 that the phenomenon of misidentification still exists, with a minimum error rate of 5%. This is due to the false peak kurtosis caused by the noise inside the signal. If this error cannot be effectively eliminated, it will limit the fault degree recognition rate in the next step. To solve this problem, error fault samples of inner race are taken as examples to further observe the multi kurtosis spectrum. Figure 7 shows four sets of multi kurtosis spectrum that incorrectly select the optimal kurtosis location.

**Figure 8:**
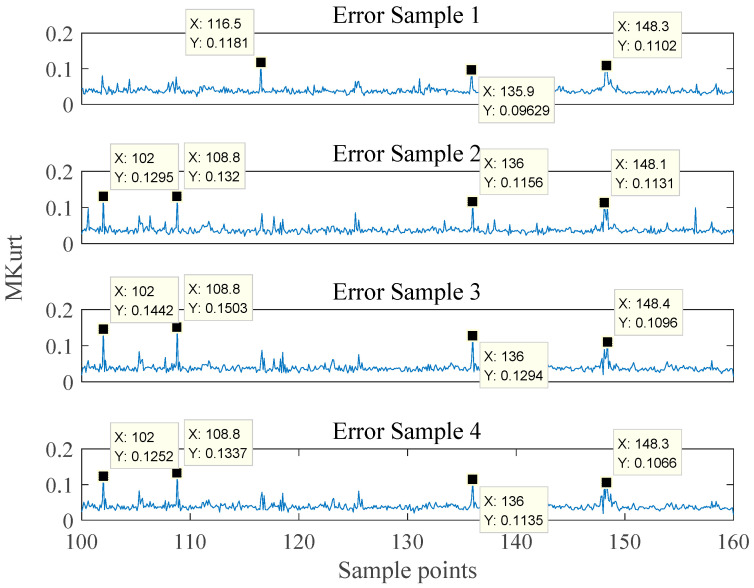
Multi kurtosis spectrum with the incorrect optimal kurtosis location.

As can be seen from the Figure 8, there are still obvious peaks at the ideal position of 148. However, it is not the global maximum when compared with the sharp peak at other locations. The MOMEDA algorithm selects the optimal kurtosis position based on the single kurtosis. False impulse makes the ideal peak position unable to be the maximum from time to time. Compared with peak value of the wrong position, peak value of the ideal position has not only a larger peak value, but also a wider sideband. An improved method called MOMLMEDA is proposed based on this feature in this paper.

**Figure 9:**
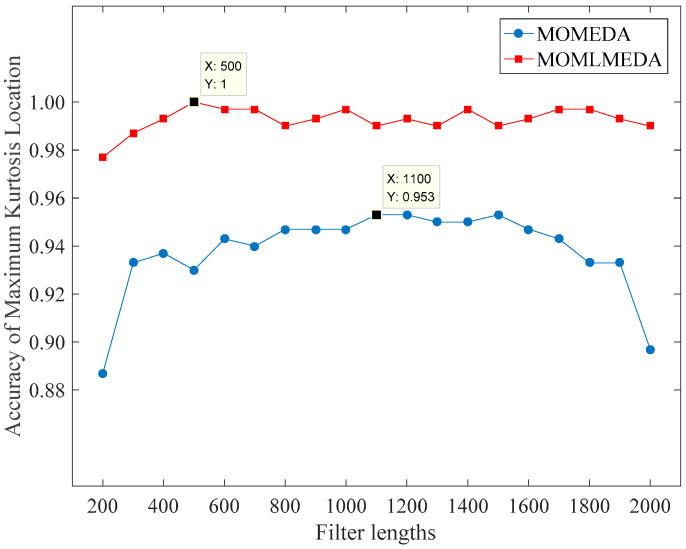
Test result under different lengths of filter via MOMLMEDA.

It takes the maximum local mean as the selection criterion of the optimal kurtosis position, effectively avoids the influence of false kurtosis peak. Five peak positions are selected, and then the average values in the local range of the peak are calculated. The location with the largest local mean is the optimal kurtosis location. The same samples were tested by this MOMLMEDA and the test result is shown in Figure 9.

**Figure 10:**
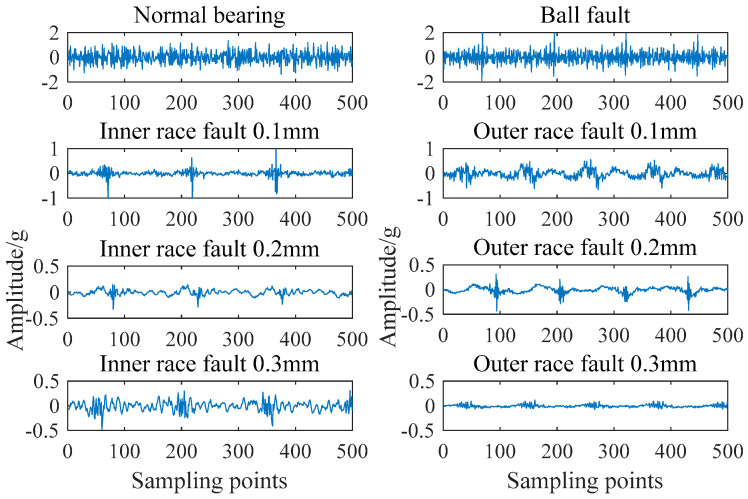
Filters of different bearing signals designed by MOMLMEDA.

Compared with the traditional MOMEDA, MOMLMEDA not only has higher accuracy in selecting the ideal kurtosis location, but also effectively shortens the filter length required for high accuracy and greatly reduces the calculation time. As can be seen from the Figure 9, when the filter length is 500, the accuracy can reach 100%. So, the filter length is set to 500. The designed filters are shown in Figure 10.

**Figure 11:**
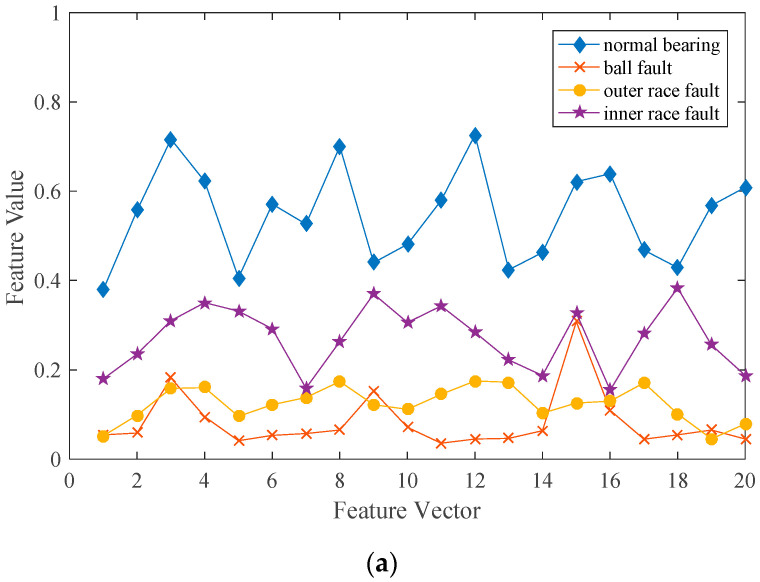

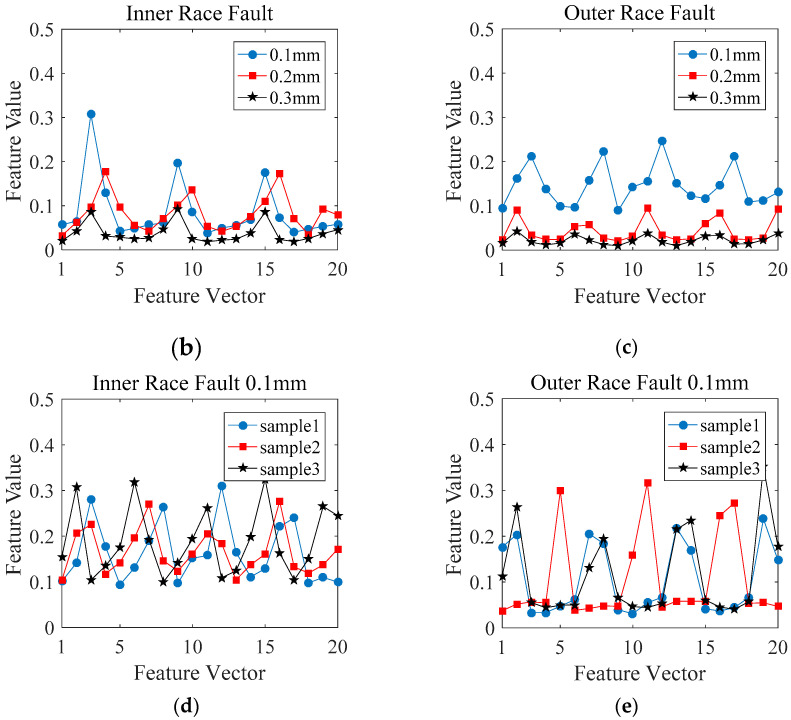
Comparison of coarse-grained feature vectors between different fault signals: (**a**) Different types of bearing signals, (**b**) Different degrees of inner race fault, (**c**) Different degrees of outer race fault, (**d**) Same degree of inner race fault, (**e**) Same degree of outer race fault.

As shown in Figure 10, there are obvious periodic differences and amplitude differences in inner race fault signals and outer race fault signals. There are also some differences in the amplitude between the original signal and the ball fault signal, which are similar before. These differences can be used to distinguish ball faults. Therefore, the optimal filter based on MOMLMEDA algorithm can be used as the source of fault features to effectively distinguish the signals with different fault degrees. In order to express these differences more intuitively and reduce the scale of classification neural network, the filter is coarsened. 500 sampling points are divided into 20 groups and the mean value of each group was calculated. Vibration signals can be represented by vectors, the length of each is 20. The coarse-grained feature vectors of different inner race faults and outer race faults are shown in Figure 11.

From (a) in Figure 11, it can be found that different types of signals have obvious differences in amplitude characteristics, especially normal signals and ball fault signals. Although there is little difference in the magnitude between inner and outer fault, it can also be seen that the periodic characteristics of the two faults are obviously different from (b) and (c) in Figure 11. The interval between the peaks of the same type of fault signal is basically the same. The feature values of different fault degrees are obviously different. This means that this feature can better classify different types of faults and degrees of faults. However, it can be seen from graph (d) (e) that the filter features have temporal characteristics. Due to different signal samples start at different time nodes, the filter can contain three to four peaks. If the feature vectors are directly used as the input of the traditional neural network, it will seriously affect the recognition rate of the whole network.

LSTM is a kind of RNN, which is used to process sequential data. It not only considers the temporal problem, but also assigns the weights of far and near time features reasonably. It solves the influence of temporal characteristic on features more perfectly. We introduce it into bearing fault diagnosis based on coarse-grained feature of filter. A LSTM network for bearing fault classification is constructed based on the classical examples in MATLAB.

**Figure 12:**
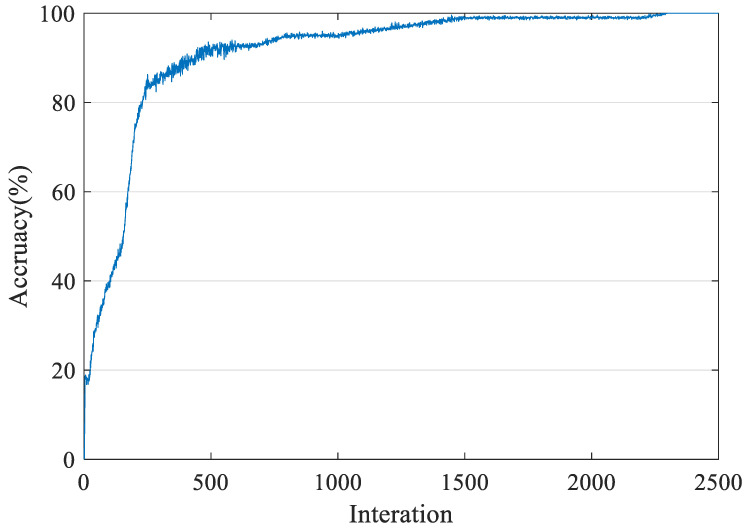
Training Process of LSTM.

The LSTM network for sequence-to-label classification includes sequence input layer, LSTM layer, full connection layer, soft max layer, and classification output layer. The size of input layer is set to 20, which is consistent with the length of feature vector. The size of the full connection layer is set to eight, which is consistent with the number of data types. The number of LSTM hidden layers is set to 50. The total number of samples is 800, which is divided into two categories: training samples and test samples. The number of samples in each category is 400. First, we put the training samples into the designed LSTM network for training. The training process is shown in Figure 12.

**Figure 13:**
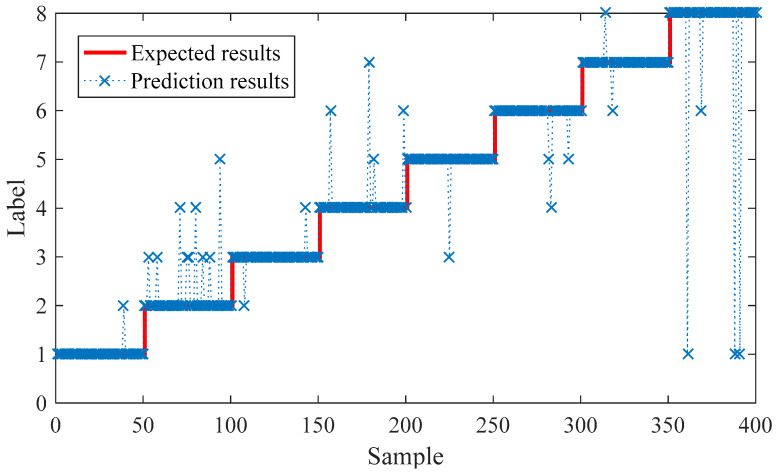
Prediction result based on MOMLMEDA and LSTM.

As shown in Figure 12, the self-recognition rate of training samples can reach 100% after approximately 1600 iterations. Then, the test samples are put into the trained neural network for testing. The test results are shown in Figure 13.

As can be seen from Figure 13, 26 out of 350 test samples had errors in recognition results, and the overall recognition rate was 93.5%. The test results show that the proposed method can effectively distinguish the signal of normal bearing, the inner race fault, the outer fault, and the ball fault. Furthermore, the signals with different fault degrees of inner race and outer race can be further distinguished.

In order to further verify the improved effect of MOMLMEDA relative to MOMEDA and the superiority of LSTM network in processing signal recognition with temporal characteristic, several groups of comparative algorithm experiments are carried out in this paper. Each group used the same original data.

Comparing group 1 and group 2 in Table 2, it can be seen that the recognition rate is increased by 4%. This proves that MOMLMEDA can obtain better fault features than MOMEDA. Comparing group 2 and group 3–5 in Table 2, it can be found that LSTM network has better classification ability of feature with temporal characteristic. This can prove that the proposed fault diagnosis combinatorial algorithm based on MOMLMEDA and LSTM is effective.

## 5. Conclusions

In order to realize the accurate diagnosis of bearing faults of different types and states, a bearing fault health monitoring method based on filter features designed by MOMLMEDA and LSTM is proposed in this paper. Firstly, aiming at the problem that MOMEDA is disturbed by noise, a MOMLMEDA algorithm is proposed. By calculating the local mean kurtosis, the false impulses generated by a single position is eliminated and the accuracy of optimal kurtosis location selection is improved. Secondly, the optimal filter is designed, and the coarse-grained mean feature is extracted based on the optimal kurtosis location. This feature contains better periodic and amplitude features, which can effectively characterize different fault types and degrees. Then, aiming at the interference of temporal problem existed in feature vectors, a classification network based on LSTM is constructed. The extracted mean feature vectors are set as the input of classification network, and the faults are accurately classified by training the network.

In this paper, eight kinds of signals including normal signals, ball fault signals, three kinds of inner race fault signals, and three kinds of outer race fault signals are collected, each of which has 100 samples. The total number of samples is 800, half for training networks and half for testing. The recognition rate of the proposed method is up to 93.50%. Compared with some other algorithms, this method shows better superiority. This proves that the bearing fault method proposed in this paper is effective.

The method presented in this paper can effectively distinguish between normal bearing, ball fault, inner race fault, and outer race fault. Furthermore, the signals with different fault degrees of inner race and outer race can be further distinguished. At present, due to the limitation of experimental materials, only the classification of ball fault has been completed. The classification of fault degree of ball faults will continue in the next stage of research.

## Figures and Tables

**Table 1:** Description of experimental data.

Bearing State	Fault Size (mm)	Sample Label
Normal	0	1
Inner race fault	0.1	2
	0.2	3
	0.3	4
Outer race fault	0.1	5
	0.2	6
	0.3	7
Ball fault	0.1	8

**Table 2:** Comparative algorithm experiments.

Number	Method	Number of Errors	Recognition Rate
1	MOMEDA&LSTM	42	89.50%
2	MOMLMEDA&LSTM	26	93.50%
3	MOMLMEDA&SAE	147	63.25%
4	MOMLMEDA&BP	221	44.75%
5	MOMLMEDA&SVM	210	47.50%
